# Effect of a Six-Week Intermittent Fasting Intervention Program on the Composition of the Human Body in Women over 60 Years of Age

**DOI:** 10.3390/ijerph17114138

**Published:** 2020-06-10

**Authors:** Przemysław Domaszewski, Mariusz Konieczny, Paweł Pakosz, Dawid Bączkowicz, Ewa Sadowska-Krępa

**Affiliations:** 1 Faculty of Physical Education and Physiotherapy, Opole University of Technology, 45-758 Opole, Poland; p.domaszewski@po.edu.pl (P.D.); p.pakosz@po.edu.pl (P.P.); d.baczkowicz@po.edu.pl (D.B.); 2 Institute of Sport Sciences, the Jerzy Kukuczka Academy of Physical Education, 40-065 Katowice, Poland; e.sadowska-krepa@awf.katowice.pl

**Keywords:** intermittent fasting, time-restricted feeding, MMSE, ABI, body composition

## Abstract

The objective of this research was to determine the effectiveness of intermittent fasting (IF) in reducing body fat and lowering body mass index. An additional objective was to determine the feasibility of applying IF in overweight women over 60 years of age, which was assessed by the ratio of subjects who resigned from the diet plan. This study included a group of 45 women over 60 years of age. The intervention in the experimental group involved complete abstinence from food intake for 16 h per day, from 20:00 p.m. to 12:00 a.m. (the next day). The results demonstrated that the body weight in the subjects in the experimental group (EXP) group decreased by almost 2 kg and this decrease was visible in the remaining parameters related to body fat mass. The skeletal muscle mass did not change significantly, which indicates an actual decrease in the fat mass. The proportion of subjects who did not succeed in following the prescribed diet plan was 12%. The application of intermittent fasting in female subjects over 60 years of age resulted in positive changes in body composition. Time-restricted feeding (TRF) was characterized by a lower resignation rate compared to other diets involving intermittent fasting.

## 1. Introduction

The aging process is an inevitable stage of human ontogeny. Currently, in developed countries, a significant extension of life expectancy can be observed among individuals in the post-working period. This is largely related to the improvement of living conditions, access to better medical care and the increasing role of diet and nutrition, which are central to the growth of life expectancy [1,2]. At the same time, statistics indicate that overweight/obesity continues its relentless global rise, with the number of people with excess body weight reaching >2 billion, ~30% of the world population. The Global Burden of Disease Group reported in 2017 that “since 1980, the prevalence of obesity has doubled in more than 70 countries and has continuously increased in most other countries” [3]. The problem of excess body weight applies to all age groups; however, it is most clearly visible in children and seniors. Important factors that contribute to negative age-related changes in body composition of seniors are insulin resistance [4] and progressive loss of muscle and bone mass [5]. In addition to the negative impact on physical health, excess weight is likely to adversely affect mental health. The body mass index (BMI) in older people is related to, among other things, frailty syndrome, which has a negative effect on the general approach to life [6]. Patients with frailty syndrome are reported to have a lower potential for coping with stress and responding to incidents in life. Thus, such people are more susceptible to various adverse health and psychological consequences (falls, infections, disability, obesity and death). In studies involving dietary and supplementary interventions in groups of seniors, it has been noted that seniors are incapable of following nutritional recommendations for extended periods of time, which very often makes it impossible to provide a reliable interpretation of the results [7,8]. Another significant problem in maintaining a healthy body weight in the elderly population is a lack of adequate physical activity [9]. The decrease in physical activity in people over 60 is often related to the incidence of cardiovascular disease. One of the common diseases significantly limiting physical activity is intermittent claudication, which can directly indicate atherosclerosis and can be indirectly recorded during ankle–brachial index (ABI) measurements [6,10].

In addition to regular physical activity, the ability to maintain a steady body mass is based on the ability to maintain an adequate diet. Under the circumstances of an existing excess body weight, increased physical activity may, however, lead to an overload of the osteoarticular system. This consequently requires that dietary interventions be undertaken first. There are several basic strategies and diets aimed at reducing excess body weight. The most common approach is based on a systematic daily restriction in caloric intake, which involves a 10% to 30% reduction in the intake of calories in relation to the actual energy demand [11]. This approach is very effective in weight reduction, but for most people, the ability to estimate the caloric value of meals seems to be too challenging. In addition, the inability to eat at will results in progressive frustration, which usually results in abandoning diet plans and a return to old habits, directly leading to a yo-yo effect [12]. As a result of the difficulties associated with the caloric restriction diet approach, there has been an increase in the application of programs involving intermittent fasting (IF). IF programs demonstrate a comparable effectiveness in lowering body fat [13], an increased susceptibility to insulin [14], a reduction of excess body weight [15], an improvement of the lipid profile [16] and a decreased risk of cardiovascular diseases [17]. The studies of intermittent fasting that have been conducted on animals and humans have also suggested a positive impact of IF programs on extending life expectancy [18], reducing the risk of cancer [19], reducing inflammatory diseases and reducing the oxidative stress that can lead to significant damage to cell structures [20].

Diet plans involving temporary restriction of food intake include intermittent periods of food intake and complete abstinence from foods. Currently, the most popular types of IF diets include either the complete [21] or partial limitation of caloric intake (by approximately 75–90% compared to energy requirements) [13] for two days per week (known as the 5:2 protocol). Another strategy is based on alternating caloric restriction (by approximately 75%) days with unlimited calorie diet days (known as ADF; alternate-day fasting) [22]. The time-restricted feeding (TRF) diet, which is the subject of this study, offers another interesting intermittent fasting strategy. This method consists of a daily diet program in which the hours of fasting and the so-called eating windows are pre-determined, lasting for 16 and 8, 18 and 6 or 20 and 4 h, respectively [23]. The 16 to 8 h protocol used in this study is considered as one of the most effective TRF because this fasting and feeding time seems to prevent adverse metabolic diseases without altering caloric intake [24]. The periods of fasting involve complete elimination of food accompanied by a permitted intake of liquids with very low or zero calorie content. During the eating window, one can eat at will. For these reasons, the aim of this research was to determine the effectiveness of the TRF eating plan in reducing body fat and lowering the body mass index. An additional aim was to determine the feasibility of TRF in people of post-working age as assessed by the degree of resignation during an experiment that lasted six weeks.

## 2. Materials and Methods

The experimental study involved a group of 45 non-smoking women over 60 years of age (65 ± 5 years, height 161.2 ± 6.7 cm, body mass 72.4 ± 12.6 kg) who belonged to a seniors’ association. The admission program for the present experiment was preceded by an information and education campaign, including a lecture on diet and healthy lifestyle delivered to the members of the seniors’ association. The subjects willing to participate in the experiment were randomly divided into two groups: a control group (CON = 20; age = 66 ± 4.7) and an experimental group (EXP = 25; age = 65 ± 4.0). The task for the control group was to follow an eating plan based on their previous habits. A weekly, detailed analysis of the diet consumed by the subjects demonstrated that all subjects observed a mixed diet with calorific value suited to their energy demands. The intervention in the EXP group involved completely abstaining from food for 16 h a day, from 20:00 p.m. to 12:00 a.m. (the next day). The study participants were also asked to maintain their normal physical activity for the duration of the entire experiment. From the statements provided by the subjects in the EXP group regarding their ability to follow the guidelines of the experiment, 22 subjects in the experimental group completed the full six-week program. The data for the subjects in the EXP group were excluded from the analysis if, in the final statement, the number of days for which the requirements of the six-week diet were not observed exceeded 10% of the total days designed for the study.

The subject data were collected two days before launching the experiment and data collection was repeated two days after completing the diet program.

The following research tools were applied in the study:

- An analysis of body composition and BMI was performed using a SECA mBCA 515 (seca GmbH & Co. KG, Hamburg, Germany) analyzer with eight electrodes. According to the guidelines stated by the National Institutes of Health, weight status was classified into four categories: underweight (BMI ≤ 18.5 kg/m^2^), normal weight (BMI between 18.5 and 24.9 kg/m^2^), overweight (BMI between 25 and 29.9 kg/m^2^) and obese (BMI ≥ 30 kg/m^2^).
- An assessment of mental state with regard to cognitive abilities was conducted using the Mini–Mental Status Examination (MMSE). The Mini–Mental State Examination, or Folstein test, is a 30-point questionnaire that is used extensively in clinical and research settings to measure cognitive impairment. It is used to estimate the severity and progression of cognitive impairment and to follow the course of cognitive changes in an individual over time. The following four cut-off levels should be employed to classify the severity of cognitive impairment: no cognitive impairment, 24–30; mild cognitive impairment, 19–23; moderate cognitive impairment, 10–18; and severe cognitive impairment, ≤9 [25].
- Measurement of the ankle–brachial index (ABI) was employed as a basic tool in the evaluation of peripheral arteries and cardiovascular risk in general practice. The American College of Cardiology and American Heart Association [6] recommend the application of ABI measurements in assessing the hazard of atherosclerosis. The ABI is a simple, cheap and non-invasive tool that correlates well with the degree of peripheral artery disease (PAD) [10]. The ABI result is calculated by dividing the systolic blood pressure measured in the arterial conduits at the level of the ankle by the systolic blood pressure measured in the brachial artery—LL, LR, RL, RR (Table 1):
  LL—Left ankle systolic blood pressure/left brachial systolic blood pressure;
  LR—Right ankle brachial systolic blood pressure/left brachial systolic blood pressure;
  RL—Left ankle systolic blood pressure/right brachial systolic blood pressure;
  RR—Right ankle brachial systolic blood pressure/right brachial systolic blood pressure.

The normal values are taken in the range of 0.9–1.15. The ankle–arm index of <0.9 indicates ischemia and <0.5 occurs in case of critical limb ischemia.

Inclusion criteria for participation in the experiment:

1. Age +60 years old.
2. Ability to understand instructions and active participation in the tasks, MME > 23 p.
3. Lack of medical contraindications to participate in the experiment and a positive result tested by ABI.
4. Agreement to participate in the research and a declaration of respect for the IF guidelines.

All participants of the study signed an informed consent form. The study was approved by the Bioethics Committee of the Chamber of Physicians and the study was conducted in accordance with the guidelines described in the Helsinki Declaration for research involving humans as well. All tests were carried out in the physiological laboratory of the Opole University of Technology.

### Statistical Analysis

The collected data were subsequently subjected to statistical analysis by application of Jamovi 1.1.9 software (The jamovi project (2020).jamovi (Version 1.2) [Computer Software]. Retrieved from https://www.jamovi.org). Due to the lack of normality of the distributions and homogeneity of the variances of the analyzed variables, nonparametric analysis tools were applied in the present analysis. To determine the level of the significance of the differences, the non-parametric Wilcoxon test was used to determine the dependencies between the samples. Repeated Measures ANOVA analysis was used to determine interactions between groups.

The research was also supported by the size of the Cohen’s d effect. The sample size was calculated to be at least 20 participants on the assumption to detect medium Cohen’s d effect size of 0.5 or larger, power 80% and a 5% (two-sided) significance level.

## 3. Results

Table 1 contains the descriptive statistics of the body composition parameters determined by the SECA system ABI and MMSE test. On the basis of these data, subjects in both the experimental group and control group had an average BMI value above 25 kg/m^2^, which means that they were overweight. In both groups, the results of the initial measurements indicated no difference in body weight (approximately 70 kg) between the groups, whereas the examination performed after the experiment demonstrated a difference (approximately 2 kg) in favor of the experimental group.

Based on the MMSE assessment of the mental state, it was found that all subjects demonstrated an absence of signs of cognitive impairment and dementia. The average value of MMSE points was 28 in the experimental group and 28 in the control group, both before and after the experiment.

The ABI results demonstrated that none of the subjects had an index below 0.9, which indicated the absence of significant limb ischemia.

Table 2 presents the results of the significant differences analysis between the experimental and control groups at the two measurement timepoints. A statistically significant difference (*p* ≥ 0.001) in the relationship between the results of initial and final tests in terms of the decrease in BMI values and weight value, in the experimental group compared to the control group, were observed. The same level of significance was obtained with regard to the differences between the initial and final tests in terms of the relative fat mass value, absolute fat mass value. The skeletal muscle mass did not change significantly, which indicates a real decrease in the fat mass.

Table 3 presents the results of the analysis of group interactions between individual variables in the experiment. The analysis showed statistically significant interactions at the level of *p* ≤ 0.01 within the three examined variables: BMI value, relative fat mass value and weight value.

## 4. Discussion

The common problem of obesity among seniors leads to the frequent application of diet programs. Despite the advantages of typical, restrictive diets based on calorie intake, research has proven that some diets also cause secondary physiological changes that reduce the capabilities of further reduction in body weight. This adaptive response to calorie restriction results in increased appetite [26], reduced physical activity [27] and hormone level fluctuations promoting fat deposition and the reduction of muscle tissue [28].

The adverse changes that accompany diets and result from long-term restrictions in calorie intake may, however, be normalized or reduced by applying periods in which energy supply is balanced to meet the demands of the body. The use of intermittent fasting promises a metabolic advantage over a long-term, constant restriction in calorie intake and may prove to be a more effective strategy of weight loss. Despite the meta-analysis of clinical trials by Seimon et al. in 2015 [29], which did not confirm this mechanism, the results of this study indicate the considerable efficiency of the TRF diet program (16:8 h) in women over 60 years of age. The majority of the experiments carried out with regard to the intermittent fasting program have proven the effectiveness of IF in weight reduction, with results ranging from 2.5% to even 9.9% weight reduction [30]. The scope and dynamic characteristics of dietary changes form a very diverse and personal issue that is dependent on the type of the diet plan, the duration of the experiment and the initial body weight of the subjects. This study also confirmed the effectiveness of intermittent fasting in achieving weight reduction. Following the six-week program, the women in the experimental group reported a reduction in body weight of nearly 2% compared with the increase of body weight by nearly 1% in the control group during the same time period. However, the most important change found in this work as a result of the TRF program was a statistically significant reduction in body fat by nearly 1.6%, which can be converted into a decrease in fat mass by 1660 g while maintaining lean mass at an almost unchanged level. For comparison, in the control group, the body fat mass increased by over 1000 g (over 1%) after six weeks, which was accompanied by a lean mass decrease of 0.66%.

Researchers also point out the important fact that even very effective diets are often not feasible in clinical application due to the significant difficulties in maintaining them over extended periods of time. The reasons for giving up diets are often associated with unrealistic eating restrictions resulting in the feeling of hunger and overall dissatisfaction [31]. In previous research on other forms of intermittent fasting, a very high index (in the range of up to 40%) of diet program abandonment was reported [32]. Such high ratios prompted statements from which it could be assumed that IF diets were not practical in clinical application [31]. However, this study confirmed the findings of dietitians and indicated that the TRF 16:8 program was much easier to maintain and, thus, it could perform well as an important tool for treating obesity in clinical scenarios. In the experimental group, three participants were not able to stick to the diet until the end of the experiment. The oral interview with the subjects made it possible to establish that only two people considered the 16:8 diet to be difficult to follow. This was reported to be related to the relatively short fasting period projected within the TRF diet. The diets involving longer periods of abstaining from foods often lead to overeating after the period of fasting, which is one of the main limitations to the application of IF diets. Due to the relatively short time without eating, the TRF protocol (16:8) was relatively better suited to the normal daily routine and did not seem to prompt overeating after the periods designed for fasting. According to the research on consciousness, the everyday habits of older people and the hazards of frailty syndrome, diet is strongly correlated with well-being and better performance of the cardiovascular system [6]. However, at this moment the number of studies regarding the influence of intermittent fasting on the cardiovascular system is very limited. One of the few studies carried out in this area confirmed the positive effect of IF on risk factors for cardiovascular disease [33].

The 16:8 protocol of TRF provides an interesting alternative to traditional diets; however, it requires further long-term studies involving larger experimental groups, with differentiation in terms of sex and age, for confirmation.

## 5. Limitations

The application of the SECA mBCA 515 analyzer in research is characterized by high accuracy and repeatability of the results; however, it is a type of test that is applied to indirectly assess body composition and may be subject to measurement error. Moreover, in this work, research subjects were exclusively women and the effects of IF diets should also be differentiated with regard to sex. This statement is confirmed by the results of the study by Heilbronn et al. [34], which indicates a better metabolic response of male subjects to intermittent fasting diets. Season could have been another factor that influenced the results of the experiment. The reduction of physical activity in autumn and winter could have contributed to the adverse changes in the body composition of women in the control group. In future studies, a bigger sample size and an accurate measurement of physical activity of the subjects could be crucial and thereby taken into consideration.

## 6. Conclusions

The application of intermittent fasting in older women resulted in positive changes in body composition. Time-restricted feeding is characterized by a lower ratio of diet resignation in comparison to other forms of fasting involving periodic abstinence from food

### Recommendations

The research shows that IF can be an alternative to traditional calorie restriction diets.

## Figures and Tables

**Table 1:** Descriptive statistics of ankle–brachial index (ABI) results in the experimental and control groups.

	EXP		CON	
	First Trial	Second Trial	First Trial	Second Trial
BMI Value	28.99 ± 5.18	27.70 ± 5.10	26.99 ± 4.20	27.61 ± 4.65
Relative fat mass value	42.62 ± 6.09	41.04 ± 5.97	41.72 ± 4.72	42.87 ± 4.35
Absolute fat mass value	30.36 ± 8.72	28.70 ± 8.63	29.38 ± 8.15	30.39 ± 8.00
Fat-free mass value	39.58 ± 4.91	39.87 ± 4.77	40.28 ± 5.95	39.82 ± 6.10
Skeletal muscle mass value	17.67 ± 2.69	17.83 ± 2.66	17.90 ± 3.47	17.67 ± 3.52
% Body total water	44.10 ± 4.20	44.70 ± 4.31	44.00 ± 3.44	43.6 ± 3.19
Weight value	69.93 ± 12.48	68.57 ± 12.39	69.66 ± 12.93	70.21 ± 13.08
Height value	155.39 ± 4.59	155.39 ± 4.59	160.42 ± 5.94	160.42 ± 5.94
ABI LL	1.16 ± 0.10	1.16 ± 0.09	1.15 ± 0.07	1.13 ± 0.08
ABI LR	1.18 ± 0.10	1.18 ± 0.08	1.15 ± 0.08	1.14 ± 0.08
ABI RL	1.18 ± 0.10	1.18 ± 0.09	1.19 ± 0.09	1.16 ± 0.10
ABI RP	1.19 ± 0.11	1.19 ± 0.10	1.18 ± 0.09	1.16 ± 0.08
MMSE	28 ± 1.94	28 ± 1.84	28 ± 1.84	28 ± 1.78

**Table 2:** Wilcoxon test results between two measurement timepoints (before/after the experiment) in the experimental and control groups.

	EXP			CON		
	Statistic T	*p*	COHEN’S D	Statistic T	*p*	COHEN’S D
BMI Value	252.00	0.001	1.5167	156.00	0.190	−0.344
Relative fat mass value	232.00	0.001	0.9372	112.00	0.023	−0.443
Absolute fat mass value	253.00	0.001	−1.4797	117.50	0.031	−0.434
Fat-free mass value	85.00	0.187	−0.2110	280.00	0.180	0.208
Skeletal muscle mass value	93.50	0.291	−0.1773	287.00	0.137	0.207
% Body total water	31.5	0.053	−0.461	146.0	0.121	0.285
Weight value	219.50	0.001	1.0920	187.50	0.523	−0.198
ABI LL	28.00	0.688	−0.0858	118.50	0.001	0.638
ABI LR	54.50	0.776	−0.0330	93.00	0.060	0.366
ABI RL	38.00	0.624	−0.0221	154.00	0.003	0.520
ABI RR	54.00	0.950	0.0272	82.50	0.063	0.390
MMSE	6.00	0.149	0.3882	6.00	0.149	0.334

**Table 3:** Repeated measures ANOVA test results between the two study groups.

Caption	F	*p*	η^2^*_p_*
BMI Value			
Group	0.62	0.434	0.012
Time	2.83	0.108	0.001
Time/Group	21.28	0.001	0.010
Relative Fat Mass Value			
Group	0.10	0.751	0.002
Time	0.46	0.503	0.000
Time/Group	18.37	0.001	0.017
Absolute Fat Mass Value			
Group	0.08	0.781	0.001
Time	136.09	0.001	0.333
Time/Group	0.29	0.591	0.001
Fat-Free Mass Value			
Group	0.04	0.833	0.001
Time	0.10	0.758	0.000
Time/Group	1.96	0.168	0.001
Skeletal Muscle Mass Value			
Group	0.00	0.969	0.000
Time	0.07	0.787	0.000
Time/Group	1.79	0.188	0.001
Weight Value			
Group	0.04	0.850	0.001
Time	1.63	0.207	0.000
Time/Group	9.01	0.004	0.001
% Body Total Water			
Group	0.08	0.785	0.001
Time	3.03	0.088	0.002
Time/Group	13.95	0.001	0.011
